# Neurochemical Alterations in Sudden Unexplained Perinatal Deaths—A Review

**DOI:** 10.3389/fped.2018.00006

**Published:** 2018-01-25

**Authors:** Nazeer Muhammad, Muhammad Sharif, Javeria Amin, Riffat Mehboob, Syed Amir Gilani, Nargis Bibi, Hasnain Javed, Naseer Ahmed

**Affiliations:** ^1^COMSATS Institute of Information Technology, Wah Cantonment, Pakistan; ^2^Research Unit, Faculty of Allied Health Sciences, University of Lahore, Lahore, Pakistan; ^3^University Institute of Physical Therapy, Faculty of Allied Health Sciences, University of Lahore, Lahore, Pakistan; ^4^Department of Computer Sciences, Fatima Jinnah Women University, Rawalpindi, Pakistan; ^5^Department of Microbiology and Molecular Genetics, University of the Punjab, Lahore, Pakistan; ^6^Medical School, University of Verona, Verona, Italy; ^7^Faculty of Health Sciences, University of the Punjab, Lahore, Pakistan

**Keywords:** sudden infant death, sudden perinatal death, stillbirth, neuropathology, sudden intrauterine death, neurochemicals

## Abstract

Sudden unexpected perinatal collapse is a major trauma for the parents of victims. Sudden infant death syndrome (SIDS) is unexpected and mysterious death of an apparently healthy neonate from birth till 1 year of age without any known causes, even after thorough postmortem investigations. However, the incidence of sudden intrauterine unexplained death syndrome (SIUDS) is seven times higher as compared with SIDS. This observation is approximated 40–80%. Stillbirth is defined as death of a fetus after 20th week of gestation or just before delivery at full term without a known reason. Pakistan has the highest burden of stillbirth in the world. This basis of SIDS, SIUDS, and stillbirths eludes specialists. The purpose of this study is to investigate factors behind failure in control of these unexplained deaths and how research may go ahead with improved prospects. Animal models and physiological data demonstrate that sleep, arousal, and cardiorespiratory malfunctioning are abnormal mechanisms in SIUDS risk factors or in newborn children who subsequently die from SIDS. This review focuses on insights in neuropathology and mechanisms of SIDS and SIUDS in terms of different receptors involved in this major perinatal demise. Several studies conducted in the past decade have confirmed neuropathological and neurochemical anomalies related to serotonin transporter, substance P, acetylcholine α7 nicotine receptors, etc., in sudden unexplained fetal and infant deaths. There is need to focus more on research in this area to unveil the major curtain to neuroprotection by underlying mechanisms leading to such deaths.

## Introduction

In the first year of life, the most frequent type of death is “Crib death,” “Cot death” commonly termed as “sudden infant death syndrome” (SIDS). Among every 1,700–2,000 births approximately, one baby gets affected (1). Numerous inherited abnormalities, such as morphological substrates for SIDS–sudden intrauterine unexplained death syndrome (SIUDS), were detected, mainly represented by variations of cardiac conduction system just like accessory pathway, abnormal resorptive degeneration, and hypoplasia/agenesis of the vital brainstem structures. The National Child Health Institute and Human Development has expressed that SIDS is a developmental issue and it takes its root from the fetal development (2). The neuropathological examination plays a significant role in the death investigation procedure. However, just some limited reviews have sufficiently analyzed the neurological substrates, albeit even subtle anomalies of the autonomic nervous system can measure the dysfunctions in the fundamental functions, prompting sudden and unexpected death (3, 4). In-depth examination results, performed at the University of Milan, Lino Rossi Research Center, have added to recognize the area and the nature of these anomalies, normally observed in both SIUDS and SIDS. External risk factors, for example, alcohol, maternal smoking, and drug abuse are identified to be the potential contributors of SIUDS and SIDS (5) while environmental pollution such as insecticides and pesticides has also been reported recently (6).

## Sudden Infant Death Syndrome

**Figure 1:**
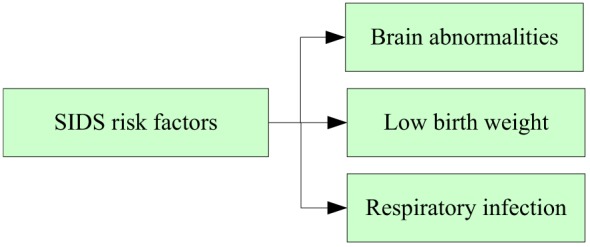
Sudden infant death syndrome (SIDS) risk factors.

In a number of these infants, the cerebrum portion that controls the arousal and breathing from sleep is not yet mature enough to work appropriately. Preterm births and intrauterine growth restrictions can cause repressed cognitive development and chronic infarctions. Low immunological development and postnatal sleeping positions are responsible for major respiratory distress. In this section, we have discussed the risk factors for SIDS. Figure 1 is derived from Filiano and Kinney hypothesis (7) and shows risk factors contributing to SIDS.

### Neuropathology of SIDS

**Figure 2:**
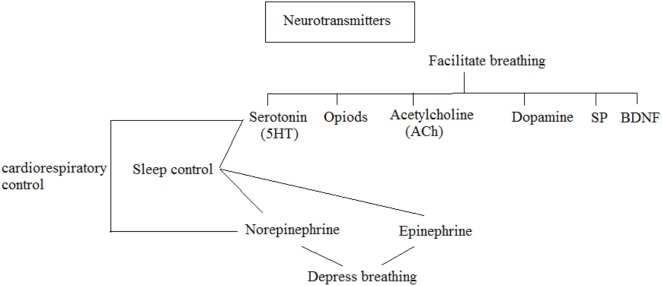
Few neurotransmitters in normal fetus and infants.

The major focus on cerebrum anomalies in SIDS victims for a physiological investigation demonstrates cardiopulmonary abnormalities and sleep arousal dysfunction. A typical pathway of these abnormalities at the level of brainstem, where these control functions including ventilation pathways, cardiac rhythm, and pathways for sleep/arousal. Neuropathological basis of SIDS, as proposed to be the major risk factor and needs more neurochemical investigation (7). Research on the neurochemical abnormalities of SIDS victims was started in the 1980s (8). Some neurotransmitters and their functions in a normal infant or fetus are shown in Figure 2.

For instance, acetylcholine (ACh) and serotonin [5-hydroxytryptamines (5-HT)] were found to facilitate breathing (9) while epinephrine (Epi) and norepinephrine (NE) depressed breathing (10). Moreover, it was demonstrated that Epi, NE, and 5-HT were additionally required in the organization of sleep (11). Dopamine was observed to be required in stimulating breath while the peptide neuromodulator substance P (SP) (12, 13), endogenous opioids (14), and derived brain growth neurotrophic factor (BDNF) (15) were observed to be required in the focal control of breath. The neuropathology abnormalities identified in the SIDS brainstem (16) are summarized in Table 1.

**Table 1:** Summary for the identification of all neuropathology abnormalities in the sudden infant death syndrome (SIDS) brainstem.

Reference	Enzyme, transmitter, or receptor	Level of brainstem	SIDS cases	Results
(17)	An immunohistochemical method involving tyrosine hydroxylase	Diencephalon, basal ganglia, midbrain, pons, and medulla oblongata	37	In SIDS, changes in basal ganglia can be induced *via* repeated ischemia or chronic hypoxia but can be associated with developing a neuronal system to the upper cardiorespiratory control
(18)	5-Hydroxytryptamines (5-HT) and 5-hydroxyindoleacetic acid	High-performance liquid chromatography and Raphe obscure and PGCL	35	SIDS was related with lower TPH2 and 5-HT levels, consistent with a deficiency of medullary 5-HT disorder
(13)	Immunohistochemical expression and substance P (SP)	Neuromodulator	20	SP localized in fiber structures, with low to high densities
(19)	^3^H-nicotine	16 brainstem nuclei	27	In the brainstem alcohol and smoking adversely affect 3 H-nicotinic binding
(20)	α7 and β2 Nicotinic acetylcholine receptors (nAChRs)	Rostral medulla and pons	46	SIDS infants have a genetic defect acquired in the molecular regulation
(21)	γ-Aminobutyric acid	Medulla	24	SIDS may essential to include therapeutic agents that target more than one neurotransmitter system
(22)	1A (5HT1AR)	Rostral medulla	67	In SIDS cigarette smoke and prone sleeping exposure support serotonergic brainstem system
(23)	Serotonergic (5-HT)	Respiratory nuclei and medulla	16	An outcome demonstrates that increased neurochemical preliminary evidence that supports boy’s vulnerability to SIDS
(24)	Interleukin-2 and cytokine	Cardiorespiratory- and sleep/arousal pathophysiology	18	The neuro-molecular disequilibrium results in the delicate molecular balance producing dysfunction in brainstem centers and disturbed homeostasis
(25)	Pro-BDNF, rh-BDNF, and TrkB	Rostral medulla	67	In the brainstem provides abnormal expression of rh-BDNF, TrkB, and pro-BDNF receptor protein of SIDS and non-SIDS infants
(26)	Pontine Kolliker–Fuse nucleus and orexin receptors	Raphe nuclei and locus coeruleus	28	KF neurons detection only 20% of SIDS

### ACh Receptor

Smoking in pregnancy fundamentally increases morbidity and perinatal mortality. It is presently the vital autonomous and modifiable risk factor adding to the sudden newborn child death disorder (SIDS) (27). The more convincing hypothesis for the connection among SIDS and smoking is that nicotine alters the vital breathing patterns and defensive reactions to hypoxia in sleeping (28). A lessened anxiety reaction intensifies hypoxia and apnea (29). The impacts of nicotine are interceded *via* its activation of very particular nicotinic cholinergic receptors (nAChRs) that are available in the carotid physiques and in the serious brainstem cores, for example, the core of single tract and locus coeruleus (30). At these locales, nAChRs add to the cholinergic adjustment of arousal and breathing. Interference with the nAChRs functions on the presumed basis of negative nicotine reactions (31). Disturbing equilibrium among arousal and ventilatory responses could intensify respiratory failure in sleeping duration. Postnatal exposure to smoke tobacco during early stages is related to increase in the number of sicknesses in repository, pulmonary impaired function, and SIDS events. It is additionally connected through reduced (32) cognitive working and attention deficits in youth. Nicotine, the main neurotoxic segment of tobacco smoke, actuates its activities *via* binding to nicotinic acetylcholine receptors (nAChR). The immunohistochemical expression of nAChR subunits α_2_, α_3_, α_4_, α_5_, α_7_, α_9_, α_1_, and α_2_ in medulla brainstem was analyzed in a piglet model after postnatal nicotine exposure (33). Table 2 describes the ACh receptor abnormalities identified in the SIDS brainstem.

**Table 2:** Summary for the identification acetylcholine receptor abnormalities in the sudden infant death syndrome brainstem.

Reference	Receptor	Samples	Results
(34)	nAChR	Procedure of all animal from National Institutes of Health Care	Calcineurin activation and reduced intracellular calcium by L-type channels
(34)	Neuronal nicotinic acetylcholine receptors (nAChR), α_7_, β_2_	Rats	The existence of nicotine (10 M) in hypoxic insult secured a subpopulation
(35)	Nicotinic acetylcholine receptors, β_2_^+/+^ mice	Animals were used from the National Research Center	Modulate β_2_-nAChRs to the survival of infant brain cells
(36)	Nicotinic cholinergic receptor (nAChR)	Feminine rats	Reduced nAChR expression in dopaminergic areas in the duration of adolescence
(31)	Nicotine impairs breathing	Age-matched wild-mutant mice deficient the subunit β_2_ nAChR gene	The nAChRs are vital in breathing in the duration of sleeping and are important for the ordinary improvement in the mechanisms of arousal
(33)	Nicotine and preBotzinger complex	Medullary slice	Nicotinic acetylcholine receptors (nAChRs) activation improved the tonic synaptic excitatory input to inspiratory neurons
(37)	Nicotinic acetylcholine receptors (nAChRs)	The animals used were an adult male, age-matched	nAChRs with β*_2_* contribute activity in REMS, NREMS, and the promoting effect of stress

### Serotonin 5-HT Neurotransmitter

**Figure 3:**
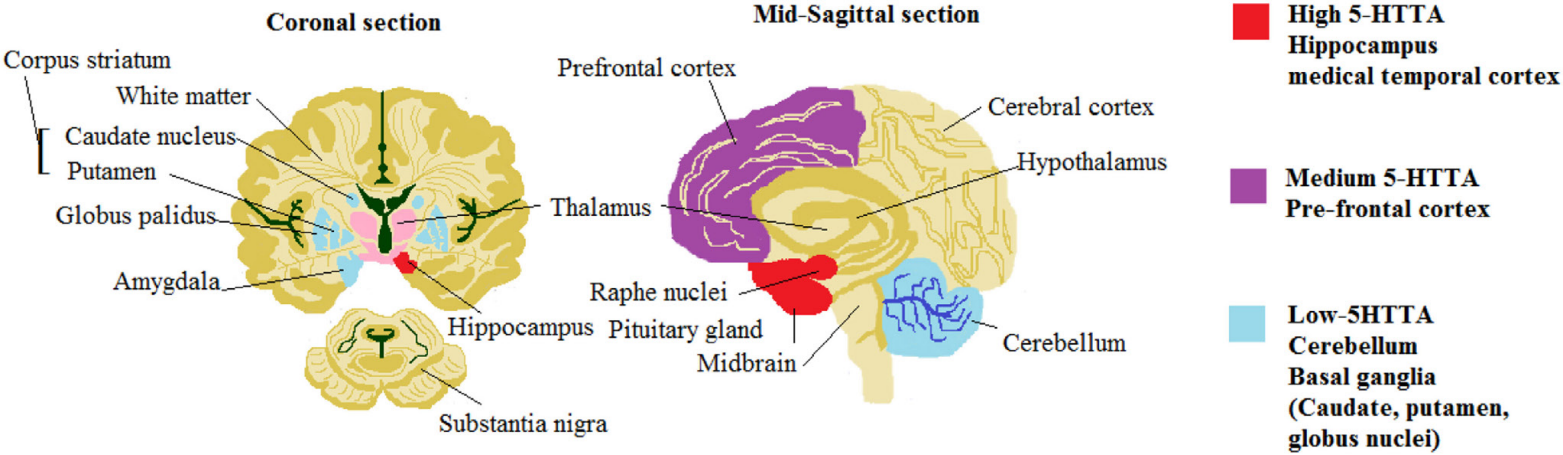
Role of serotonin 5-hydroxytryptamines (5-HT) neurotransmitter.

In the brain development, serotonin 5-HT neurotransmitter performs a central role in stress reactivity, mood regulation disorders of psychiatric risk factors and subsequently signaling in 5-HT in the early stage have complicated implications on mental health and behavior over the life span. It takes part in the intercession of cognition, arousal, mood, cerebral blood flow and motor activity. It regulates cardiovascular and cardiorespiratory function, chemosensitivity, thermoregulation, arousal, and pain (38). Figure 3 shows the role of serotonin 5-HT. SIDS victims have been found to have reduced levels of brainstem serotonin (5-HT) and tryptophan hydroxylase 2 (TPH2) but retain producing 5-HT neurons. TPH2 is cerebrum particular enzyme that translates tryptophan into 5-HTP, which is then transformed over into 5-HT *via* DOPA decarboxylase.

Due to the disturbance in 5-HT medullary levels that outcomes in deadly dysfunction of these dependent sodium-pacemaker neurons regulated *via* projections of 5-HT from the Raphe and additional Raphe cores (39). We assumed that alteration might be brought about by contrasts in serotonin transporter mRNA expression and 5-HT1A receptor in mind regions included in the control of feelings, memory, and additionally in areas controlling the focal serotonergic tone (40). Table 3 summarizes all the serotonin 5-HT neurotransmitter abnormalities identified in the SIDS brainstem so far.

**Table 3:** Summary for the identification of serotonin 5-hydroxytryptamines (5-HT) neurotransmitter abnormalities in the sudden infant death syndrome.

Reference	Year	Method	Sample	Results
(41)	2014	5-HT	45 mice	In the hypothalamus gene expression, it minimizes the 5-HT2A receptor
(42)	2014	Tryptophan hydroxylase 2 (TPH2), 5-HT	Group of mice	TPH2^−/−^ mouse is a useful model in the new medications searches for depression
(43)	2014	Serotonin (5-HT) and oxytocin (OXT)	4 healthy males	In the amygdala effects of OXT on 5-HT1A within the subgenual cortex can be mediated *via* induced effects occurring of OXT
(44)	2015	5-MT injection	Animals from National Organization of Health	5-Methoxytryptamine shows that the CYP2D-catalyzed different pathway synthesis of serotonin
(45)	2017	5-HT_7_	Mice	5-HT_7_ brain receptor–ERK system performed a vital role in the adaptation of stress formation
(46)	2017	5-HT_4_R	24 healthy participants and 3 woman	In the association’s differences, 5-HT_4_R binding between negative, positive, and neutral word categories did not statistically reach
(40)	2014	5-HT_1A_ and mRNA expression	Adult rat	Serotonin transporter mRNA reduction shows variants in polymorphic individuals with depression at the higher risk

### Low Birthweight and Respiratory Tract Infection

The sudden infant deaths are multifactorial, where low birthweight has been reported major risk factor for SIDS (47). Viral respiratory infections are mainly responsible for the occurrence of sudden death. Mild level of respiratory viral infection was observed by investigators in cases of sudden death infants up to 80% (48).

## Sudden Intrauterine Unexplained Death Syndrome

**Figure 4:**
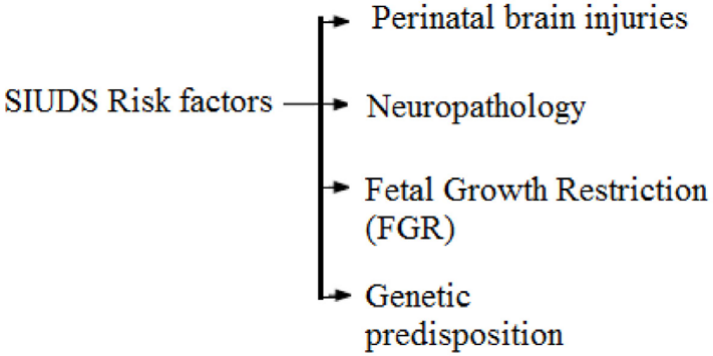
Sudden intrauterine unexplained death syndrome (SIUDS) risk factors.

Risk factors for SIUDS are shown in Figure 4. Perinatal brain injuries may occur due to trauma during pregnancy, birth asphyxia, and postnatal accident (48).

Fetal growth restriction (FGR) is a significant difficulty of pregnancy showing a fetus that does not develop to maximum capacity because of pathological compromise. FGR influences 3–9% of pregnancies in high-salary nations and is the main source of perinatal mortality. Placental insufficiency is the key reason for FGR, bringing about chronic fetal hypoxia. This initiates hypoxia of an adaptive fetal reaction of cardiovascular yield redistribution to support indispensable organs, involving the mind and is in result called cerebrum sparing. In spite of this, it is currently apparent that cerebrum sparing does not guarantee normal cerebrum growth in limited development fetuses (49). A brief summary of SIUDS is mentioned in Table 4.

**Table 4:** Summary for the identification of abnormalities in the sudden intrauterine unexplained death syndrome.

Reference	Year	Methods	Tested sample	Results
(50)	2014	Fetal growth restriction (FGR)	Rats, guinea pigs, rabbits, and sheep	FGR is related with minimizing brain volume and altered structure, cortical volume, and decreased total myelination that deficits cells number
(51)	2014	Magnetic resonance imaging (MRI), corpus callosum, and intrauterine growth-restricted (IUGR) fetuses	173 IUGR fetuses	Further explored corpus callosum to predict anomalous neurodevelopment risk in pregnancies
(52)	2014	NRG1-IVNV	41 cases	Development of human neocortical provides expression of quantitative NRG1 isoform
(53)	2017	Cerebral palsy	Therapeutic candidates	Injury to developing the brain caused by the cerebral palsy
(54)	2017	Perinatal hypoxia	Humans and animals	In the FGR hypoxia is a vital problem in fetal–maternal medicine
(55)	2017	Human amnion epithelial cells (hAECs)	Mouse model	hAECs release trophic factors
(56)	2003	Diagnosis of IUGR, respiratory distress syndrome, intraventricular hemorrhage, and necrotizing enterocolitis	Newborn infants	Increased IUGR with prematurity and represent a vital risk factor in women when present with labor preterm
(57)	2015	Ultrasound appearance of brain volume and cortical development in fetuses	20 fetuses	Brain volume smaller in IUGR fetuses, with accelerated or normal cortical maturation as depicted with the examination of postnatal MRI, can be described by 3D prenatal ultrasound
(58)	2015	HbF and BCL11A	3 patients	It highlights the significance of using hematopoietic-specific methods when trying to target therapeutically BCL11A

Numerous neurodevelopmental issues of cognitive and motor function have their origins in the antenatal period. Fetal suboptimal growth is probably a key variable underlying altered cerebrum growth. FGR is related with perinatal death, preterm birth and, for survivors, an expanded risk of sensory and motor neurodevelopmental deficits, learning and cognitive impairments, and cerebral palsy. The implementation of the neuroprotective treatments can just happen in light of careful characterization of the abnormalities in brain growth that increases because of FGR, first require the identification of newborn children at most serious risk for the impairment of neurodevelopmental secondary to fetal poor development. Eighty pregnancies end up in termination following detection of an abnormal fetal, neonatal death, or stillbirth, describing no less than eight thousand cases per annum, and there are more than 500 unexplained baby and youth deaths every year. In these circumstances, the posthumous examination is frequently required to decide reason for death, set up implications for relatives, and direct administration of future pregnancies (55).

Fetal growth restriction is generally viewed as a risk for perinatal cerebrum injury with intraventricular hemorrhage (IVH), yet clinical reviews record altered outcomes with elevated, decreased, or unaltered rates of IVH in FGR newborn children contrasted with suitably developed counterparts. Placental insufficiency with anomalous umbilical artery Doppler was connected to the occurrence of IVH. While considering that cerebrum sparing is a characteristic reaction to placental chronic hypoxia, it is not amazing that changes in blood flow to the cerebrum might be both characteristics of the clinical seriousness of FGR, and related with impairments of neurodevelopment. The adaptive reaction of cerebrum sparing requires remodeling of the fetal cerebrum flow that can be diagnosed *via* Doppler ultrasound as a reduced pulsatility record in the cerebral arteries. At the point when a vast cohort of children was isolated into weekly birth interims, it was found that rates of IVH in FGR were significantly lower versus non-FGR newborn children born at 28 weeks, proposing a defensive impact of development limitation, however, that IVH rates elevated significantly in late-FGR preterm births >34 weeks. This outcome has been confirmed by a recent review demonstrating that IVH was common in late-FGR preterm babies contrasted with suitably developed newborn children. That concern the finding of late preterm births, >34 and <37 weeks, represent most preterm births, and occurrence of preterm births is expanding (59, 60).

Sudden intrauterine unexplained death syndrome is multifactorial and polygenic condition. Although several genetic factors have been reported as cause of SIUDS but defining a specific genetic aberration at this stage is often a challenging issue due to limited phenotype–genotype correlation (61). In addition, genetic anomalies in under developed phenotypes are rarely investigated. Several studies have reported through whole genome sequencing the importance of neurodevelopmental and ion exchange pathway genes (*ARHGAP35, BBS7, CASZ1, COL2A1, CRIM1, DHCR7, HADHB, HAPLN3, HSPG2, MYO18B, RYR1*, and *SRGAP2*).

## Conclusion

A brainstem abnormality is suggested to be the main underlying etiological factor in SIUDS and SIDS victims. Alterations in certain neurotransmitters such as ACh receptor, serotonin 5-HT neurotransmitter, SP, and brain-derived neurotrophic growth factor (BDNF) are identified in the SIDS and SIUDS, which have vital roles in chemosensation and cardiorespiratory control leading to these sudden deaths. However, further studies are suggested to investigate more into this serious life threatening events.

## Author Contributions

All the authors have contributed equally in writing the manuscript.

## Conflict of Interest Statement

The authors declare that the research was conducted in the absence of any commercial or financial relationships that could be construed as a potential conflict of interest. The reviewers BA and ÖÖ and the handling editor declared their shared affiliation.
